# Sigma B regulated motility and chemotaxis in Bacillus cereus

**DOI:** 10.1099/mic.0.001659

**Published:** 2026-01-27

**Authors:** Linda Huijboom, Marcel Tempelaars, Sjef Boeren, Erik van der Linden, Mehdi Habibi, Reza Shaebani, Tjakko Abee

**Affiliations:** 1 Food Microbiology, Wageningen University, Wageningen 6708WG, The Netherlands; 2 Biochemistry, Wageningen University, Wageningen 6708WG, The Netherlands; 3 Physics and Physical Chemistry of Foods, Wageningen University, Wageningen 6708WG, The Netherlands; 4 Department of Physics, Universität des Saarlandes, Saarbrücken 66123, Germany

**Keywords:** bacterial flagella, bacterial dynamics, run-and-tumble, stress response

## Abstract

This study describes an alternative role of the general stress response (GSR) regulated by Sigma B, via the two-component system RsbKY, which is methylated via RsbM, in motility regulation for the peritrichously flagellated, motile, foodborne pathogen *Bacillus cereus*. Using a set of Sigma B-related mutants, we found reduced surface spreading on low-agar plates for all mutants compared to the WT of *B. cereus* ATCC 14579. The GSR mutants still contained flagella similar to WT in the samples taken from the edge of colonies with reduced surface spreading. Using cell trajectory analysis of selected mutants and WT, we found that the Sigma B-controlled Hpr-like phosphocarrier *bc1009* mutant had a reduced duration of the run phase, resulting in an overall lower persistence and coverage of the surface area over a given time. Indeed, prolonged incubation of low-agar ‘swimming’ plates resulted in full coverage by all GSR mutants, indicating functional motility, but reduced efficiency. Proteome analysis of samples from low-agar plates showed overall lower expression levels of motility-related proteins and, in particular, significantly lower values for proteins related to the C-ring, involved in the regulation of the run-and-tumble motion of bacteria. The *bc1009* mutant showed an additional downregulation of a subset of methyl-accepting chemotaxis proteins, involved in the activation of the key chemotaxis regulators CheA and CheY. We propose a new chemotaxis model, in which CheA and CheY are still key regulators, but an additional regulatory role on the run-and-tumble motion is proposed for the Sigma B-regulated Hpr-like protein Bc1009 via the unique two-component system RsbKY.

## Introduction

*Bacillus cereus* is a Gram-positive, peritrichously flagellated, motile, endospore-forming, foodborne pathogen that can be found in a wide variety of food products, including grains, dairy, spices, meats and vegetables [1]. It can cause both emetic disease, via the ingestion of foods containing the heat-stable toxin cereulide, as well as diarrhoeal disease, which is characterized by ingestion of alive cells and/or spores surviving the gastric passage followed by outgrowth and subsequent enterotoxin production of the cells [2]. Even though diseases caused by *B. cereus* are often underreported, they are still estimated to account for 1.4–12% of all foodborne illness outbreaks worldwide, and more research is needed on their ecology and lifestyle outside of the host [3,4].

Spreading and survival of *B. cereus* cells and their subsequent transmission from the environment to food products are in part related to their highly resistant spores [5], capability to produce biofilms [6] and their general stress response system [7]. The general stress response of Gram-positive bacteria, such as *B. cereus*, enables vegetative cells to increase their survival to environmental stresses, also encountered during food processing, and is regulated by the alternative sigma factor B (SigB) [8]. Initial activation of the SigB regulon is mediated by the two-component system (TCS) RsbK-RsbY. In the presence of environmental stimuli, the signal-receiving sensor kinase RsbK auto-phosphorylates its histidine residue in the H-box, initiating the transfer of a phosphate group to the RsbY phosphatase [9]. Once phosphorylated, RsbY dephosphorylates the anti-sigma factor antagonist RsbV, resulting in an increased affinity of RsbV for the anti-sigma factor RsbW. Upon forming the RsbV-RsbW complex, RsbW is released from SigB, and the SigB regulon is activated [10]. In the absence of an environmental signal, the methyltransferase RsbM methylates RsbK, which blocks the transfer of a phosphate group from RsbK to RsbY, thus leaving the system inactive [11]. Induction of SigB is related to environmental stresses such as heat [7], salt [12], H_2_O_2_, acid and ethanol exposure [13]. Although stress exposure leads to higher expression levels of SigB in *B. cereus*, (cross-)protection to lethal stresses remains limited in comparison with other Gram-positive bacteria such as *Listeria monocytogenes* [14] and *Bacillus subtilis* [15]. Notably, the membrane-associated RsbK histidine kinase contains a variety of N-terminal histidine kinase sensory domains, including CHASE3, HAMP and GAF domains, of which the last one has been implicated in small ligand/cyclic nucleotide binding and redox/light/metabolite sensing [16]. In the same work by de Been and colleagues, a genomic association was found between RsbK and CheR, a methyltransferase that methylates methyl-accepting chemotaxis proteins (MCPs). A first glimpse of the putative alternative roles of the general stress response (GSR) in *B. cereus* was recently reported by Yeak *et al.* [17], showing that a subset of SigB regulon members under the control of the SigB-regulated Hpr-like phosphocarrier protein Bc1009 had lower expression of motility-related proteins and a reduced colony size on low-agar swimming plates.

A key element in the motility of *B. cereus* is the use of its peritrichous flagella, which allow it to ‘run and tumble’ [18]. Each flagellum consists of a thin helical appendage, called the flagellar filament, that is connected via the rod and hook to a motor, composed of two stator units (MotA and MotB) [19]. The stator units engage with the C-ring, consisting of FliG, FliM and FliN/Y, which determines the rotation of the flagella either clockwise (CW) or counterclockwise (CCW), resulting in the filaments bundling together for a directed movement of the cell (‘run’) or unbundling of the filaments and tumbling of the cell (‘tumble’), respectively [20]. Motility is influenced by environmental stimuli that attract or repel cells towards a favourable or unfavourable substance, respectively, a process known as chemotaxis [21]. Environmental signals are mediated via chemoreceptors or MCPs, which activate the sensor kinase CheA. As a result, the small chemotaxis protein CheY is phosphorylated, which allows it to bind to FliN of the C-ring of the flagellar motor and triggers a switch from CCW to CW rotation of the flagella [22]. The phosphorylation state of CheY is regulated by FliY in *B. subtilis* [23], and although the homologue of FliY in *B. cereus* was found to be required for chemotaxis and a function as flagellar motor-switch protein was hypothesized, the exact function remains unknown [24].

Combining available information, including the putative alternative role of the various sensory domains in RsbK and their potential interaction with CheR(-homologue)-mediated methylation of MCPs related to chemotaxis [16] and the reduced expression of motility proteins in the *B. cereus* SigB and/or Bc1009 deletion mutants [17], we used an extended set of clean knock-out mutants to provide a deeper understanding of the role of RsbKY, SigB and Bc1009 in modulating motility and chemotaxis behaviour of *B. cereus* ATCC 14579.

## Methods

### Bacterial strains and growth conditions

ATCC 14579 was used as the WT strain and to construct all single and double mutants. Table 1 shows an overview of all mutants used in this study. An overnight culture (OC) was prepared by inoculating 5 ml tryptone soy broth (TSB) (Oxoid) in a 50-ml Greiner tube (Greiner Bio-One) with a 1 µl loop of cells from the −80 °C stock. Cultures were incubated overnight (16–18 h) at 30 °C, with continuous shaking (160–180 r.p.m.).

**Table 1.** Overview of WT and mutants used in this study

Strain/mutant	Gene no.	Description	Reference
ATCC14579	–	*B. cereus* WT strain	American Type Culture Collection
Δ*rsbK* (ery)	*bc1008*	*B. cereus rsbK* mutant with erythromycin cassette insert, *B. cereus rsbK* clean KO mutant	[9]
Δ*rsbY*	*bc1006*	*B. cereus rsbY* clean KO mutant	This study
Δ*sigB*	*bc1004*	*B. cereus sigB* clean KO mutant	[17]
Δ*bc1009*	*bc1009*	*B. cereus bc1009* clean KO mutant	[17]
Tn-*flgG*	*bc1671*	*B. cereus* transposon mutant with insertion in *flgG* (motility deficient)	[17]
Δ*rsbM*	*bc1007*	*B. cereus rsbM* clean KO mutant	This study
Δ*cheY*	*bc1627*	*B. cereus cheY* clean KO mutant	This study
Δ*cheA*	*bc1628*	*B. cereus cheA* clean KO mutant	This study

### Construction of clean mutants

Antibiotic-free in-frame (clean) deletion mutants (KO) (Table 1) were obtained via homogenous recombination using either the heat-sensitive plasmid pAUL-A as described by Warda *et al.* [25] or pKSV7 as described by Fox *et al.* [26]. For both plasmids, the target gene was amplified from genomic DNA by KAPA HIFI Hotstart Ready Mix (KAPA Biosystems, USA) using the primers listed in Data S1 (available in the online Supplementary Material). The resulting fragments were ligated in frame to either plasmid’s multiple cloning site via EcoR1 and Sal1 restriction sites. The resulting plasmids were electroporated (25 µF, 400 Ω, 1.2 kV) in a 0.2-cm cuvette using Bio-Rad GenePulser into the appropriate *B. cereus* cells and plated on either Luria–Bertani (Merck) containing 10 µg ml^−1^ erythromycin or brain heart infusion (BHI) (BD) agar plates containing 10 µg ml^−1^ chloramphenicol for pAUL-A and pKSV7, respectively. Plates were incubated at 30 °C for 24–48 h to select for transformants. Three antibiotic-resistant colonies per construct were inoculated in separate 50-ml Greiner tubes containing 10 ml of the corresponding media with antibiotics. An OC was made at 30 °C shaking at 160 r.p.m., before transferring 50 µl to 10 ml of fresh media with antibiotics and incubating overnight at 42 °C. Next, again 50 µl was transferred into 10 ml of fresh media, this time without antibiotics, and incubated at 30 °C shaking at 160 r.p.m. overnight before plating on BHI. Resulting colonies were tested for antibiotic sensitivity, and PCR analysis, followed by DNA sequencing, confirmed the correct internal in-frame deletion of the gene of interest and the lack of other mutations in the flanking regions.

### Low-agar plate motility (surface spreading)

To assess the movement of WT and mutant cells, low-agar ‘swimming’ plates were used which allow motile cells to migrate through the agar, increasing colony surface as described by [17]. The swimming plates were prepared by pouring 50 ml of TSB with 0.25% agar (w/w) into 145 mm × 20 mm round plates (Greiner Bio-One, 639102). The plates were dried in a flow cabinet for 15 min at room temperature. Mid-log cell cultures were prepared by inoculating 20 ml of sterile TSB in a 100-ml Erlenmeyer flask with the OC to an OD_600_ of 0.05. The cells were grown at 30 °C with rapid shaking (160–180 r.p.m.) until an OD_600_ of ~0.4 was reached. The mid-log cells were collected by pelleting 5 ml of the culture at 8,000 g for 5 min, discarding the supernatant and resuspending the pellet in 5 ml PBS. Next, the swimming plates were inoculated by pipetting 5 µl of the resuspended mid-log cells in the middle of the plate. The plates were dried in a flow cabinet until the droplet was absorbed and incubated at 30 °C for 21 or 48 h. After incubation, the plates were photographed, and the colony surface was determined using ImageJ [27]. Pictures were converted to 8-bit type, and the threshold was manually adjusted to detect the colony area as accurately as possible based on hue. If necessary, due to interfering reflections, the colony area was manually outlined. Next, the inner part of the plate was selected, and the particles were analysed to obtain a particle quantification of the colony area. The maximum plate size in pixels was used to obtain a surface/plate ratio and subsequently calculate the surface area in cm^2^.

### Enumeration of cells

To calculate the number of cells grown after 21 h of incubation, the cell counts of the 0.25% motility agar plates were determined. Along with the 0.25% agar plates as described in the ‘Low-agar plate motility (surface spreading)’ section, the same mid-log cultures were also used to inoculate traditional 1.5% agar plates as a control. The samples grown on 0.25% agar plates were resuspended by homogenizing the whole agar plate with 50 ml of peptone physiological salt (PPS) in a stomacher machine (Stomacher 400 circulator) at 230 beats for 1 min to obtain a 1:1 dilution. Cells grown on the 1.5% agar plates were suspended by removing the colony using a sterile spork and vortexing vigorously in a 50-ml Greiner tube containing 9 ml PPS. The resulting suspensions were diluted tenfold in PPS, and the appropriate dilutions were plated using an Eddy Jet (mode E) on BHI plates. Plates were incubated at 30 °C for 20–24 h before counting.

### Transmission electron microscopy for flagella visualization

Cells were grown on 0.25% agar plates as described in the ‘Low-agar plate motility (surface spreading)’ section at 30 °C for 21 h, and a sample was taken from the outer edge of the colony and resuspended in 500 µl of Tris buffer pH 8. The cells were pelleted for 1 min at 2,880 g and washed once with 500 µl Tris buffer. A droplet of cells was added to ionized copper TEM grids and left for 30 s before excess liquid was removed with filter paper. Cells were stained with a 2% uranyl acetate for 30 s and visualized using a JEOL 1400 plus (Wageningen Electron Microscopy Centre, Wageningen University and Research, The Netherlands) operated at 120 kV. Each sample was examined for single cells to allow room for visualization of the flagella, and a minimum of 10 cells per sample was imaged for reference.

### Cell trajectory analysis

To determine the run and tumble motions of WT and selected mutants, video recordings of cells under the microscope were made. Samples were taken from the edge of a colony grown on 0.25% agar plates as described in the ‘Low-agar plate motility (surface spreading)’ section and resuspended in TSB. Cells were visualized on 100 micron Bürker Türk slides (Cellvision) under the microscope (Zeiss) at 1,000× magnification using phase-contrast settings. Video recordings were made using an Oppo Find X3 Lite with a frame rate of 29.6 frames/sec. Subsequently, the recordings were analysed to determine the trajectory per cell along with their successive run and tumbling phases. Our tumbling detection algorithm is based on identifying the significant changes in speed *v*(*t*) and angular velocity *ω*(*t*) of the bacterium; see, e.g. [28,30]. To this aim, we determine local extrema of *v* and *ω* over time. Each minimum of *v* is surrounded by two maxima located at *t*_1_ and *t*_2_. The largest local change is chosen as the depth of the speed minimum δv, i.e. δv=max[*v*(*t*_1_)−*v*(*t*_min_), *v*(*t*_2_)−*v*(*t*_min_)]. Similarly, each maximum of ω is surrounded by two minima located at *t*_1_ and *t*_2_, from which the height δω is obtained as δω=max[*ω* (*t*_max_)− *ω*(*t*_1_), *ω*(*t*_max_)−*ω*(*t*_2_)]. The tumbling phase was determined as the time period during which the following criteria are fulfilled: (i) δv/*v*(*t*_min_)≥1, (ii) *v*(*t*)−*v*(*t*_min_)≤ δ*v*/4, (iii) The total directional change during the time interval *t*_2_−*t*_1_ exceeds √(*t*_2_−*t*_1_), and (iv) *ω* (*t*_max_)− *ω* (*t*)≤ δω. The criteria (i) and (iii) ensure that a sudden reduction of speed is accompanied by a sudden change in the direction of motion. The two other criteria determine the duration of the tumbling event [29,30]. In addition, the persistence of each strain was calculated as described by [30], used to calculate the asymptotic diffusion coefficient *D*, obtained from MSD_asymp_(*t*) = 2 Dt.

### Proteome sample preparation and analysis

Cells of WT, Δ*rsbK*, Δ*rsbY*, Δ*sigB*, Δ*bc1009* and Tn-flgG, as a control, from the outer edge of colonies grown on 0.25% agar plates as described in the ‘Low-agar plate motility (surface spreading)’ section, were collected in a 2-ml low protein-binding Eppendorf tube (Eppendorf) and resuspended in 500 µl Tris buffer pH 8. The cells were pelleted and washed twice with 200 µl ice-cold 100 mM Tris buffer before being stored at −80 °C. After defrosting, the cell pellet was resuspended in 50 µl 100 mM Tris buffer, pH 8, and sonicated on ice for three times 15 s with vortexing in between. Protein concentrations were determined using the bicinchoninic acid method [31] and set to 60 µg per 50 µl sample. The samples were further prepared for proteomic analysis using the protein aggregation capture method [32] as described by Huijboom et al., 2023 [33]. Samples were examined in biological triplicates, and each protein sample was analysed by injecting 5 µl into an nLC1000-Exploris 480 MS/MS as described before [34]. Liquid Chromatography-Mass Spectrometry (LCMS) data were analysed using the MaxQuant quantitative proteomics software package [35] and a *B. cereus* ATCC 14579 (UniProt UP000001417) database using settings as described [36]. Data from the mass spectrometry proteomics can be made available upon demand.

### Proteome data analysis

The normal logarithm was taken from protein label-free quantification (LFQ) MS1 intensities as obtained from MaxQuant. Zero ‘Log LFQ’ values were replaced in Perseus [37] by a value taken from a normal distribution using default down shift (1.8) and width (0.3) values to make sensible ratio calculations possible. In Perseus, samples were grouped, and two-sample t-tests were performed using the ‘LFQ intensity’ columns obtained with (permutation-based) false discovery rate set to 0.05 and S0 set to 1. Protein changes were deemed significant if *P*≤0.05 and ≥3 log_2_ change. Next, significant lists and volcano plots were generated in RStudio using the following packages: ‘dplyr’ [38], ‘tidyverse’ [39], ‘ggplot2’ [40] and ‘ggrepel’ [41].

### Data analysis and statistics

Data were gathered in Excel and further analysed and visualized in RStudio unless otherwise specified. After confirming normal distribution and equal variance of the data, the significance was determined using a one-way ANOVA test, and samples were considered significant if *P*≤0.05.

## Results

To investigate the potential role of the GSR in motility, we initially started with four GSR-related mutants: Δ*rsbK*, Δ*rsbY*, Δ*sigB* and Δ*bc1009*. The transposon mutant *flgG* was included as a negative control, since it lacks flagella and can subsequently be considered non-motile.

### Surface spreading

**Fig. 1.**
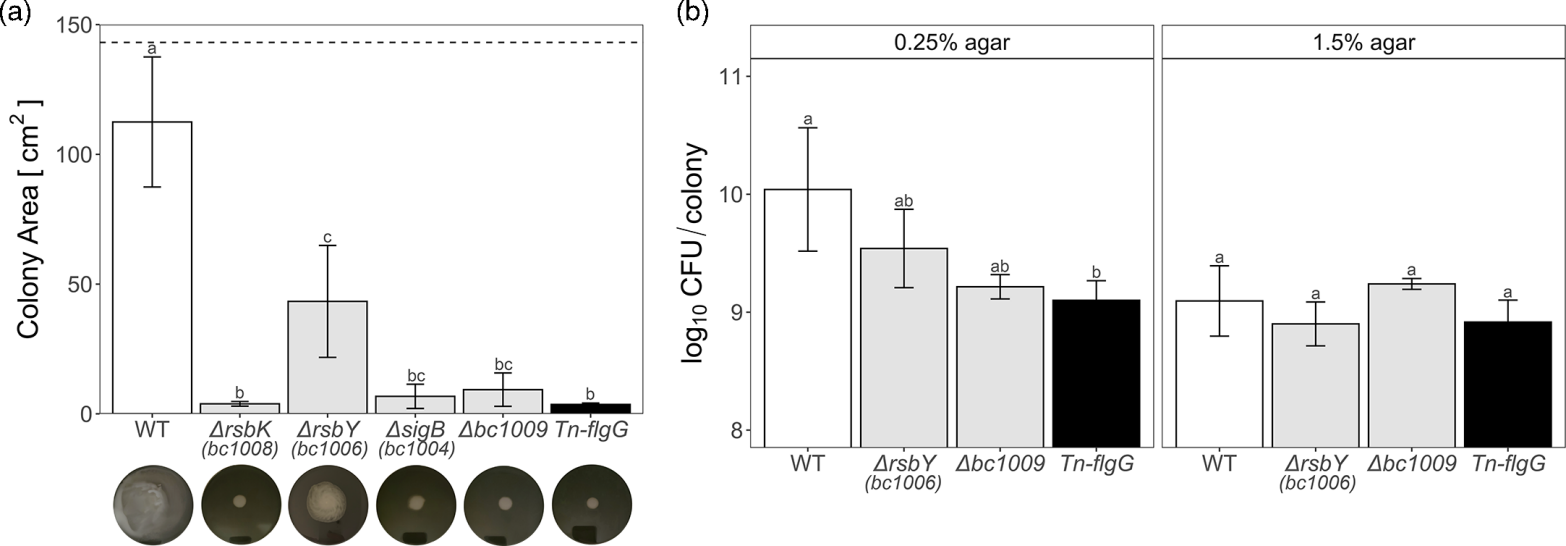
(a) Bar graph with corresponding pictures below showing the surface area of *B. cereus* ATCC 14579 (WT, white), four GSR-mutants (light grey) and Tn-*FlgG* (black) on 0.25% agar TSB plates after 21 h incubation at 30 °C. Maximum surface area is indicated by the dotted line. (**b**) Cell counts of WT, Δ*rsbY*, Δ*Bc1009* and Tn-*FlgG* after 21 h of growth at 30 °C on 0.25% and 1.5% agar TSB plates. For both graphs, *n* ≥5, and significance was determined per graph and indicated by different letters (*P*≤0.05).

The colony surface area of WT and five mutants on 0.25% agar plates was assessed as a measure of motility (Fig. 1a). After 21 h of incubation at 30 °C on TSB, the *B. cereus* WT colony had spread, reaching an average surface area of 112 cm^2^, whilst the surface area of Δ*rsbK,* Δ*sigB* and Δ*bc1009* was on average >10 cm^2^ and similar to that of the flagella deletion mutant (Tn-*flgG*). Interestingly, Δ*rsbY* showed an intermediate phenotype. No differences in surface area between the WT and five mutants were found on 1.5% agar plates (data not shown).

### Enumeration of cells

Next, the total number of cells on 0.25% agar plates and 1.5% agar plates, as a control, was determined for WT, Δ*rsbY*, Δ*bc1009* and Tn-*flgG* (Fig. 1b). Corresponding to the largest surface area on 0.25% agar plates, WT showed the highest cell counts of ~10 log_10_ c.f.u. colony^−1^, whilst the motility-deficient Tn-*flgG* mutant showed tenfold lower cell counts. Intermediate cell counts for both Δ*rsbY* and Δ*bc1009* can be observed, but these were not significantly different. Notably, cell counts of all three mutants were similar compared to WT on 1.5% agar plates of ~9 Log_10_ c.f.u. colony, corresponding with the similar colony surface areas. These results indicate that enhanced spreading correlates with enhanced growth on 0.25% agar swimming plates, whilst growth potential is similar for WT and the tested mutants on the 1.5% agar plates.

### Flagella imaging with transmission electron microscopy

**Fig. 2.**
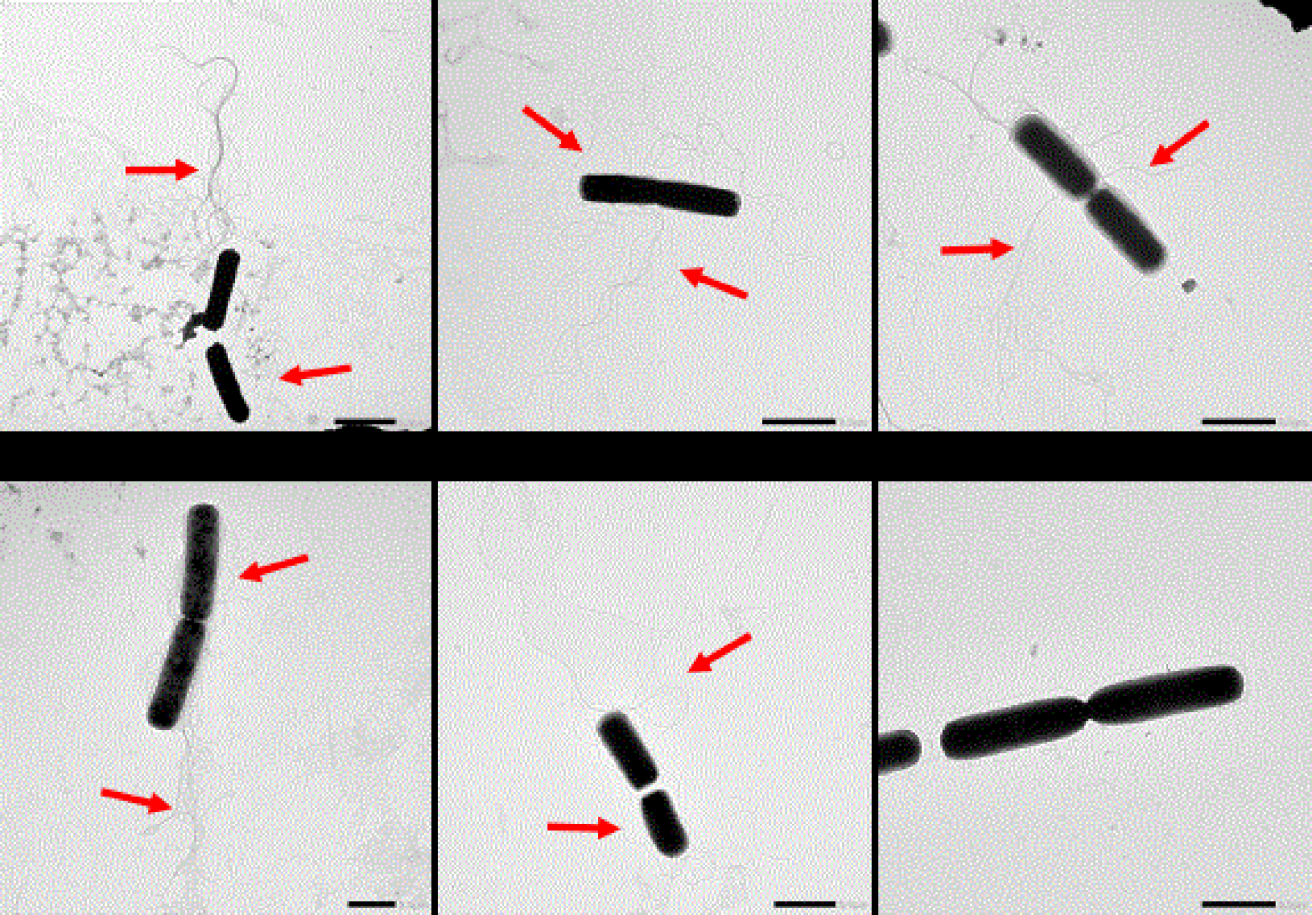
TEM images of *B. cereus* ATCC 14579 (WT), Δ*rsbK*, Δ*rsbY*, Δ*sigB*, Δ*bc1009* and Tn-*FlgG* cells taken from the edge of a colony grown on 0.25% agar TSB plates for 21 h at 30 °C. Flagella are indicated with red arrows, and the scale bar in the lower right corner of each image represents 2 µm.

As the reduced motility of the mutants may be due to the lack of flagella, the presence of flagella from cells grown on 0.25% agar plates was assessed with transmission electron microscopy (TEM). Analysis confirmed the absence of flagella in the Tn-*FlgG* mutant, whilst all four GSR-related mutants showed flagella expression comparable to WT in the samples taken from the edge of colonies with reduced surface spreading (Fig. 2). This indicates that reduced spreading/motility on the plate by the GSR-related mutants cannot be explained by loss of flagella.

### Cell trajectory analysis

**Fig. 3.**
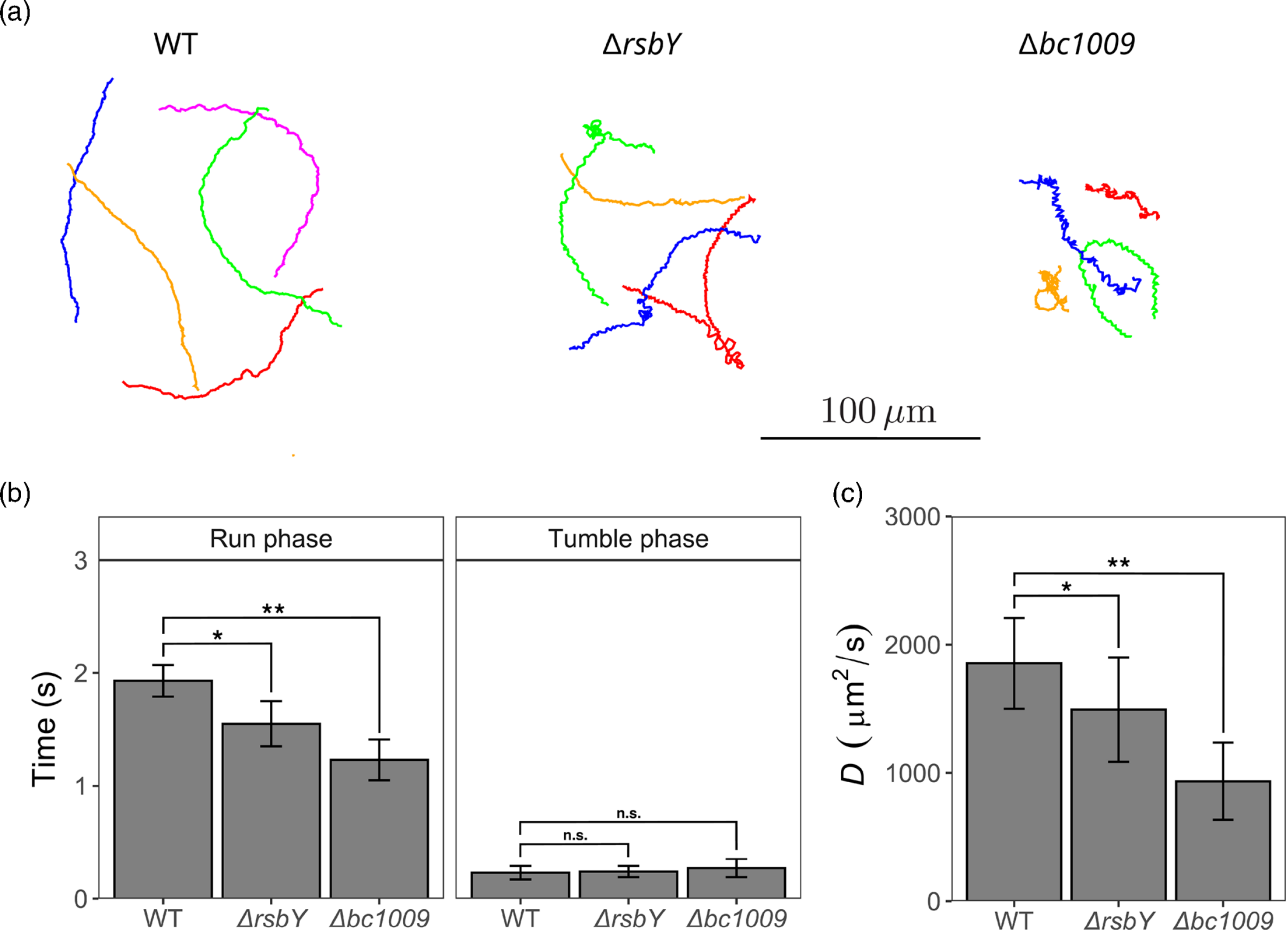
(a) Sample trajectories of WT, Δ*rsbY* and Δ*bc1009* cells during 8 s of motion. (**b**) The average duration of WT, Δ*rsbY* and Δ*bc1009* cells in the run phase and tumble phase. (**c**) The asymptotic diffusion coefficient *D* for WT, Δ*rsbY* and Δ*bc1009*. Statistical analysis in panels (b) and (c) is performed using a t-test (*P<0.05; **P<0.01; n.s., not significant).

Next, individual cells of WT, Δ*rsbY*, Δ*bc1009* and Tn-*flgG* were investigated under the microscope and traced to determine their run and tumble patterns (Data S2). As Tn-*flgG* cells were nearly immobile, with only wiggling in place without a net displacement, they were excluded from the analysis. By tracking individual cells and displaying their trajectories, a difference in their movement patterns can already be observed (Fig. 3a). The WT trajectories appear more directed compared to the trajectories of Δ*bc1009* cells, whilst Δ*rsbY* cells show an intermediate pattern. Interestingly, the average speed between WT and both mutants was similar in both the run and tumble phases (Data S3). However, a difference could be observed in the average time spent in the run phase, with WT cells remaining in this phase the longest and Δ*bc1009* cells the shortest (Fig. 3b). No difference was found regarding the time spent in the tumble phase between the WT and the two mutants. In addition, a higher persistence was found for WT cells in the run phase, which results in a higher diffusion coefficient *D* (Fig. 3c), indicating that WT cells can cover a larger area over time, especially compared to Δ*bc1009*. Overall, this indicates that although the mutants are motile, their movement appears less directional, and the duration of their run phase is shorter, resulting in a less efficient spreading compared to WT cells.

### Extended surface spreading

Further examination of the GSR-related genes in the KEGG database revealed alternative annotations for *rsbK* (*bc1008*) and *rsbY* (*bc1006*) as *cheA* (*bc1628*) and *cheY* (*bc1627*), respectively. Both *cheA* and *cheY* are known key players amongst the conserved chemotaxis genes for various organisms [42]. Interestingly, although the alternative annotations for *rsbK* and *rsbY* were found in the description of the respective genes, they are not included in the overview of the chemotaxis pathway in KEGG. Clean KO mutants of *cheA* (*bc1628*) and *cheY* (*bc1627*) were also constructed. In addition, a clean KO mutant of *rsbM* was constructed, as it is known to interact with RsbK to regulate the expression of *sigB*. All three new mutants showed reduced motility compared to WT and were similar to that of the flagella-deficient mutant after 21 h of incubation (Data S4). Notably, extension of the incubation time showed that all the GSR-related mutants displayed WT-like surface spreading after 48 h, in contrast to that of the two chemotaxis mutants (Δ*cheA* and Δ*cheY*), which remained similar to that of the non-motile flagella mutant (Data S4). Subsequent TEM imaging to confirm the presence of flagella for Δ*cheY* and Δ*cheA* showed limited to no flagella formation for Δ*cheY* and severely reduced (attached) flagella for Δ*cheA* compared to WT (data not shown).

### Proteome differences between WT and GSR mutants

As previous results indicated alterations in expression levels of motility-related proteins, we performed a comparative proteome analysis of WT, Δ*rsbK*, Δ*rsbY*, Δ*sigB* and Δ*bc1009* cells sampled from the outer edge of colonies grown on 0.25% agar plates. Overall, a general trend of lower expressed motility-related proteins could be observed for Δ*rsbK*, Δ*sigB* and Δ*bc1009* (Data S5), whilst expression of the motility-related proteins in Δ*rsbY* was more similar to WT. Particularly interesting proteins that were lower expressed in Δ*rsbK*, Δ*sigB* and Δ*bc1009* compared to WT were related to the C-ring (Table 2). From the C-ring proteins, the flagellar motor switch protein FliN (Bc1661) was significantly and profoundly lower expressed in all three mutants (log_2_ between 6 and 7.3, corresponding to 60- to 158-fold). The two C-ring proteins (Bc1662 and Bc1663) belonging to the same cluster were also remarkably lower expressed for all three mutants and significantly for both Δ*rsbK* and Δ*bc1009*. In addition to the C-ring proteins, the flagellar biosynthetic protein FliP was also significantly lower expressed in WT compared to all three mutants. Additionally, significantly lower expressed motility proteins for Δ*bc1009* compared to WT include one more flagellar motor switch protein of the C ring (Bc1629), one flagellar hook-associated protein (Bc1637), one flagellin protein (Bc1659) and five MCPs (Bc0404, Bc0422, Bc0559, Bc0576 and Bc0678). CheA and CheY were found to be not significantly lower expressed in the three mutants compared to WT.

**Table 2.** Overview of all motility-related protein abundance ratios of Δ*rsbK*, Δ*sigB*, Δ*bc1009* and Δ*rsbY*, over WT Values are indicated in log_2_ fold change (FC) and significant values are in bold. Grey highlight corresponds to significant (*P*≤0.05) values ≥3 log_2_ fold. Two-component system and methyl-accepting chemotaxis proteins are abbreviated to TCS and MCP, respectively.

Comparison			Δ*rsbK* over WT	Δ*sigB* over WT	Δ*bc1009* over WT	Δ*rsbY* over WT
KEGG ID	KEGG_group	Protein name	log_2_ FC	log_2_ FC	log_2_ FC	log_2_ FC
Bc0404	MCP	Methyl-accepting chemotaxis protein	**−2.6**	−3.8	**−4.4**	−0.7
Bc0422	MCP	Methyl-accepting chemotaxis protein	−1.8	−2.4	**−3.4**	−0.8
Bc0559	MCP	Methyl-accepting chemotaxis protein	−2.8	−2.6	**−4.0**	−0.5
Bc0576	MCP	Methyl-accepting chemotaxis protein	−1.8	−2.6	**−3.5**	−0.3
Bc0678	MCP	Methyl-accepting chemotaxis protein	−2.5	−3.0	**−4.4**	−0.5
Bc0872	Others	Cystine-binding protein	−0.1	−0.5	−0.6	−0.6
Bc1626	Stator	Chemotaxis motB protein	−2.5	−0.5	−2.5	3.4
Bc1627	TCS	Chemotaxis protein CheY	−1.3	−1.5	−3.1	−1.5
Bc1628	TCS	Chemotaxis protein CheA	−1.2	−1.6	−2.1	−0.4
Bc1629	C ring	Chemotaxis protein CheC/FliN	−3.2	−4.6	**−5.2**	−0.8
Bc1632	TCS	Chemotaxis protein methyltransferase	−0.5	−2.8	−2.9	−0.5
Bc1636	Rod and hook	Flagellar hook-associated protein 1	−0.4	−0.3	−1.1	−1.1
Bc1637	Rod and hook	Flagellar hook-associated protein 3	−3.6	−3.0	**−5.0**	−1.2
Bc1638	Flagellin	Flagellar hook-associated protein 2	**1.3**	1.4	1.0	−0.3
Bc1639	Flagellin	Flagellar protein FliS	−2.2	−2.3	−3.9	−3.9
Bc1642	Rod and hook	Flagellar basal-body rod protein FlgC	−2.0	−3.1	−3.1	0.3
Bc1644	M ring	Flagellar M-ring protein FliF	−1.4	−1.8	−2.0	−0.1
Bc1645	C ring	Flagellar motor switch protein FliG	−1.6	−1.8	−2.6	−0.6
Bc1646	Type III secretion	FliH domain-containing protein	−2.5	−2.5	−1.4	−1.2
Bc1647	Type III secretion	H(+)-transporting two-sector ATPase	−2.7	−3.9	−4.1	−0.3
Bc1651	Rod and hook	Flagellar hook protein FlgE	−1.8	−1.7	**−2.2**	−0.2
Bc1654	TCS	Chemotaxis protein CheV	−1.6	−1.7	−2.1	−0.6
Bc1657	Flagellin	Flagellin	−2.0	−2.1	−2.8	−1.3
Bc1658	Flagellin	Flagellin	−2.3	−2.7	−3.0	−2.0
Bc1659	Flagellin	Flagellin	−2.3	−2.5	**−3.3**	−1.6
Bc1661	C ring	Flagellar motor switch protein FliN	**−6.0**	**−6.2**	**−7.3**	−1.2
Bc1662	C ring	Flagellar motor switch protein FliM	**−4.3**	−4.5	**−5.7**	−0.7
Bc1663	C ring	Flagellar motor switch protein FliN	**−7.0**	−4.8	**−7.0**	−2.3
Bc1665	Type III secretion	Flagellar biosynthesis protein FliP	**−4.7**	**−4.7**	**−4.7**	−0.1
Bc2006	MCP	Methyl-accepting chemotaxis protein	−2.2	−3.4	−4.3	−0.4
Bc3385	MCP	Methyl-accepting chemotaxis protein	−4.0	−4.0	−3.0	−0.5
Bc4512	Stator	Chemotaxis MotB protein	−3.0	−2.3	**−2.5**	0.1
Bc4513	Stator	Chemotaxis MotA protein	−3.4	**−1.5**	−5.2	−0.2
Bc5009	MCP	Methyl-accepting chemotaxis protein	−4.6	−3.1	−4.8	−0.6
Bc5034	MCP	Methyl-accepting chemotaxis protein	−2.0	−2.0	**−2.6**	−0.5
Bc5065	MCP	Methyl-accepting chemotaxis protein	−4.0	−1.1	−3.1	−1.4
Bc5424	MCP	Methyl-accepting chemotaxis protein	−2.5	−2.8	−4.4	−0.4

Besides motility-related proteins, we found that the majority of significantly higher or lower expressed proteins by Δ*rsbK*, Δ*rsbY*, Δ*sigB* and Δ*bc1009* overlapped with the expression pattern of the motility-deficient mutant Tn-*FlgG* (Data S5). No differently expressed proteins (not related to motility) were found for ΔrsbK, whilst Δ*rsbY*, Δ*sigB* and Δ*bc1009* showed 14, 6 and 12 differently expressed proteins, respectively (Data S6). The 14 differently expressed proteins for the intermediate phenotype mutant Δ*rsbY* include a >200-fold higher expression of a FxsA protein (Bc4598), along with a phage protein (Bc1894), carboxypeptidase (Bc5389) and a permease (Bc3064). Significantly lower expressed proteins include four plasmid pBClin15 encoded proteins, the adapter protein MecA (Bc1490) and a cold shock protein (Bc1603). Expression of *bc1009* is *sigB*-regulated and was previously shown to be involved in the expression of a subgroup of genes [17]. The results presented in Table 3 highlight significant protein changes for Δ*bc1009* compared to WT and the other GSR mutants, including an approximate 7 log_2_ fold increase of a cystine-binding protein (Bc0402) and a 3.8 log_2_ fold increase of a branched-chain-amino-acid aminotransferase (Bc1396). Lower expressed proteins include Bc3466, a ferrichrome-binding protein, which is 4.1 log_2_ fold lower expressed, and two toxin component proteins: Bc3104 and Bc5239 (−4.8 and −5.1 log_2_, respectively).

**Table 3.** Significant protein abundance ratio changes of Δ*bc1009*/WT that are different from the expression pattern of Tn-FlgG/WT comparison Protein ratios were considered significant if the change was ≥3 log_2_ and *P*≤0.05. The abbreviation FC stands for fold change

	Protein ID	KEGG ID	Annotation	log_2_ FC	FC
**Δ*Bc1009*/WT**	Q81II5	Bc0402	Cystine-binding protein	**7.0**	**128.9**
	Q81CC5	Bc2848	Oligopeptide-binding protein oppA	**4.6**	**23.8**
	Q817M3	Bc4515	Esterase	**4.5**	**22.8**
	Q81C54	Bc2927	Prolyl endopeptidase	**4.4**	**21**
	Q81G15	Bc1396	Branched-chain-amino-acid aminotransferase	**3.8**	**13.8**
	Q81AU0	Bc3466	Ferrichrome-binding protein	**−4.1**	**−17**
	Q81CN9	Bc2713	UvrC-like protein	**−4.4**	**−20.5**
	Q815C3	Bc5241	IG hypothetical 16680	**−4.6**	**−24.6**
	Q81BP7	Bc3104	Haemolysin BL lytic component L2	**−4.8**	**−27.7**
	Q815C5	Bc5239	Enterotoxin/cell-wall binding protein	**−5.1**	**−35**

## Discussion

The combined results of the low-agar motility assays, TEM images of flagella, trajectory analysis and proteomic findings indicate an involvement of the GSR via the unique TCS RsbKY in the motility of *B. cereus* ATCC 14579. Understanding the relation between the GSR and bacterial chemotaxis is of high importance as they play a role in various biological processes such as biofilm formation, quorum sensing, bacterial pathogenesis and host infection [43]. The TEM flagella images and surface spreading after 48 h showed that the GSR mutants still have a functional motility system, albeit less efficient, as indicated by the trajectory analysis. The observed differences can likely be attributed to a difference in the regulation or structure of the motility system and reduced functioning of the chemotaxis system. Similarly, a difference in regulation is expected for the intermediate phenotype of Δ*rsbY*, which, although larger than the other GSR mutants, still showed reduced spreading compared to WT. The trajectory analysis also indicated intermediate results for Δ*rsbY* between WT and Δ*bc1009,* and protein levels resembled those of WT, and although overall lower motility proteins were found, they were not significant. When motile, bacteria alternate the faster run phases with slower tumble phases. In the trajectory analysis, the shorter duration of the run phase for Δ*bc1009* leads to (i) shorter directional movement, (ii) more tumble phases that result in a decrease in speed and (iii) more frequent changes of direction. Such motility behaviour, in combination with the lower levels of selected chemotaxis proteins in the GSR mutants, can result in a lower persistence and coverage of the surface area. In line with this, prolonged surface spreading showed that the mutants can cover the entire plate when given more time.

Both the location and number of flagella can play a role in the motility of flagellated bacteria. Peritrichous flagellation, such as in *B. cereus*, is especially important for moving over surfaces [44], and the number of flagella has been shown to play a role in the turning angle and run time of bacteria [45]. Based on our TEM images, we have no indications for a significantly different number or localization of the flagella for any of the GSR mutants. Further quantification of the flagella in future work is needed to exclude the effect of flagella abundance on the observed phenotypes.

**Fig. 4.**
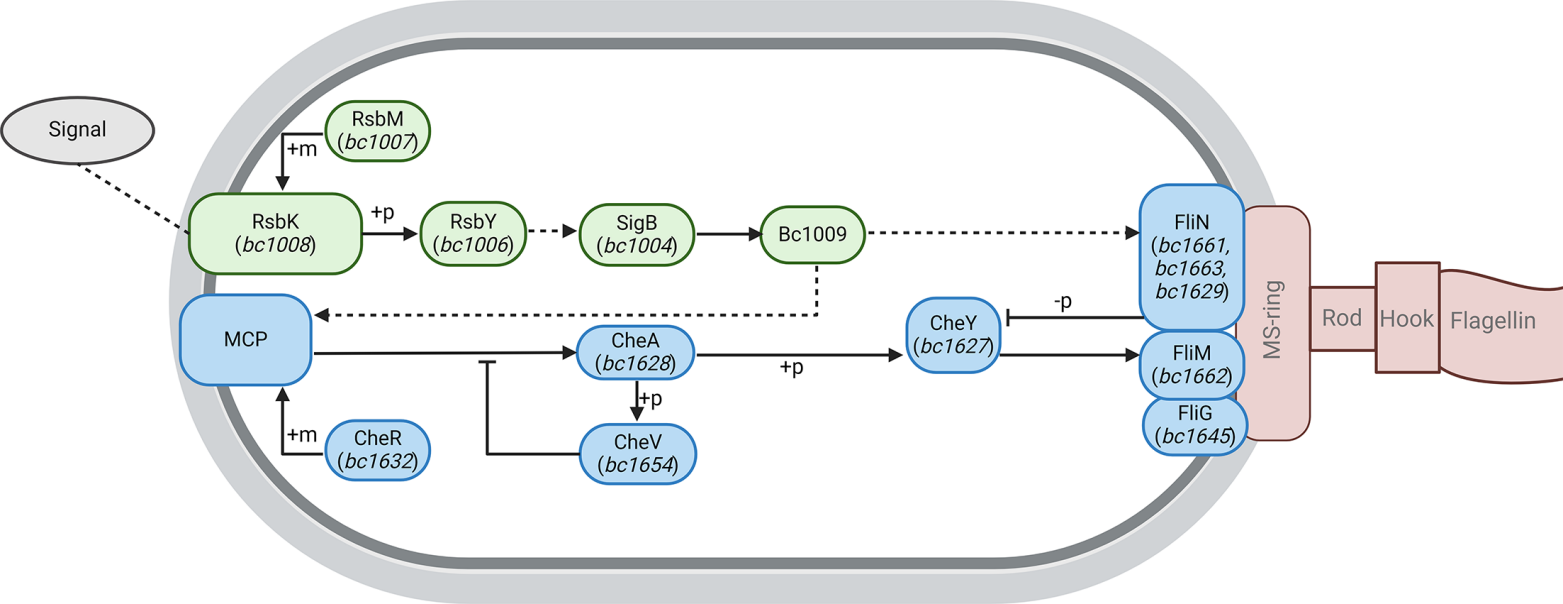
Proposed new chemotaxis model involving the GSR in *B. cereus* ATCC 14579. The following groups are indicated: proteins related to the GSR (green), proteins related to the traditional chemotaxis route (blue), flagellar unit (brown) and the cell wall/unknown signal (grey). Solid arrows indicate interactions, whereas the dotted arrows indicate a (potential) regulatory/transcriptional effect. Figure created with BioRender.com.

From the proteome analysis, we identified potential candidates involved in the adjusted regulation of the motility system. The three GSR mutants that showed reduced surface spreading all showed substantially reduced levels of proteins related to the C-ring. In the KEGG database for *B. cereus* ATCC 14579, the C-ring is composed of FliG (bc1645), FliM (bc1662) and FliN (bc1629, bc1661 and bc1663), and the FliNs are indicated to regulate CheY phosphorylation levels (Data S7). Phosphorylated CheY can bind to the C-ring to regulate the direction of the flagella rotation, but the regulation and effect of this differ greatly per species. In the extensively studied system of Gram-negative *Escherichia coli*, the binding of CheY-P to the C-ring induces CW rotation that results in tumbling of the cells. This process is regulated via CheZ, which catalyses the rate of CheY dephosphorylation [46]. However, in the Gram-positive *B. subtilis*, there is no homologue of CheZ, and binding of CheY-P to the C-ring has the opposite effect and promotes CCW rotation, resulting in smooth swimming [47]. Instead of CheZ, *B. subtilis* uses the much larger protein FliY, which also displays sequence similarities to both FliN and FliM that are a part of the C-ring [22]. For *B. cereus*, not much is known about the structure and functions of the chemotaxis proteins. The substantially lower expression of FliM and FliN proteins in Δ*rsbK*, Δ*sigB* and Δ*bc1009*, along with the reduced duration of the run phase and increased frequency of the tumble phase in Δ*bc1009*, suggests a GSR-mediated effect on the C-ring-regulated CheY phosphorylation essential for the run-and-tumble motion of bacteria. This proposed influence on the rotation direction of the flagella by the GSR via the unique two-component system RsbKY and SigB-regulated Bc1009 on the well-known chemotaxis system is depicted in Fig. 4, where Bc1009 is proposed to have a direct influence on the C-ring proteins, as evident by the significant protein differences for this mutant. As the function and mechanism of the C-ring proteins differ per species and depend on the type and number of proteins present [48], further research is required to elucidate the exact C-ring composition and function in *B. cereus*.

An additional significantly lower expressed motility-related protein in Δ*rsbK*, Δ*sigB* and Δ*bc1009* compared to the WT is FliP (bc1665), a component of the flagellar export system. As the three mutants were found to have flagella and *fliP* is located next to the *fliN* genes, it is part of the same genomic cluster which can explain the reduced protein levels. Future work should include a structural analysis of the *B. cereus* flagellar motor to elucidate whether the GSR also influences the anchorage of the rotary motors at the base of the flagella. Previous work in *B. cereus* has shown that the flagellar export system can play a role in the secretion of the toxin haemolysin BL (HBL), but is not dependent on it and instead points to an unidentified regulatory link between motility and virulence [49]. More work highlights the relation between motility and pathogenicity for *B. cereus* [50,52]. Interestingly, results obtained with Δ*bc1009* showed significantly lower levels of putative HBL lytic component L2 and a putative enterotoxin cell-wall binding protein. In addition, transcriptional repressor MogR [53,54] has been shown to regulate flagellar motility and virulence in closely related *Bacillus thuringiensis* [55]. In *B. cereus* ATCC14579, the protein encoded by *bc1655* is a hypothetical protein but contains a MogR DNA-binding domain. Furthermore, it is located on the genome next to the *cheA* regulator *cheV* (*bc1654*) and flagellar assembly genes. Smith and colleagues found MogR in *B. thuringiensis* to be essential, as they were unable to obtain a MogR deletion mutant, highlighting a function as a pleiotropic transcriptional regulator [55]. The chemotaxis regulators CheA and CheY are highly conserved throughout species and play a key role in chemotaxis [56]. Our KO mutants showed similar reduced motility as the flagella-deficient mutant, even after prolonged incubation up to 48 h. In addition, flagella synthesis was reduced for both mutants, indicating an unexpectedly larger phenotypical change that exceeds a difference in signal regulation of the run-and-tumble motions upon deletion of *cheA*/*cheY*. Therefore, in our proposed new model (Fig. 4), CheA and CheY are still key regulators needed for efficient run-and-tumble regulation. Whether the key motility regulator MogR corresponds to Bc1655 for *B. cereus* ATCC 14579 and potentially interacts with CheA/CheY remains to be elucidated, and future research, including protein–protein interactions, is required to confirm its role in the regulation of *B. cereus* motility.

Interestingly, Δ*bc1009* proteomics data showed a subset of additional differently expressed proteins compared to Δ*sigB*. Yeak and colleagues identified a subset of SigB-regulated proteins to be *bc1009* dependent, including proteins related to motility [17]. Interestingly, these proteins were not found to be lower expressed under heat shock, but only at 30 °C, suggesting a different function for *bc1009* when the *sigB* regulon is not induced. The previously identified motility proteins from cells grown in liquid culture partially overlap our own results from cells grown on low-agar motility plates, including reduced levels of two C-ring proteins [FliN (Bc1663) and FliM (Bc1662)] and three MCPs (Bc0404, Bc0576 and Bc0678). On low-agar plates, three more significant MCPs are found for Δ*bc1009,* of which two are above the 3 log_2_ fold cut-off (Bc0422 and Bc0559) and one just below (Bc5034). MCPs respond to a number of different signals in order to regulate chemotaxis, including attractants, repellents, antagonists and other environmental effectors, which regulate the degree of methylation of the MCPs via CheR [57]. This results in the phosphorylation of CheY via CheA, thus enabling CheY-P binding to the C-ring. Modulation of MCP levels via Bc1009 provides an additional pathway that may have contributed to the observed differences in spreading, next to the Bc1009 modulation of C-ring proteins, and the proposed route is depicted in Fig. 4. Similarly to CheR regulating the methylation of MCPs, RsbM can methylate and subsequently regulates RsbK, and the Δ*rsbM* mutant showed similar results to the other GSR-mutants, confirming its role in motility regulation, presumably via RsbK methylation (Fig. 4). As the MCPs are distributed throughout the genome, the overall lower expression further suggests a regulatory function for Bc1009 regarding a subset of the MCPs. *B. cereus* has various regulators that are influenced by environmental signals, such as PlcRa, Sigma 54 and CodY. Interestingly, our proteomics results indicate some overlap for Δ*bc1009* with all these regulators. PlcRa was found to play a role in cysteine metabolism and the oxidative stress response [58]. Similarly, Δ*bc1009* showed 7 log_2_ higher levels of a cystine-binding protein (Bc0402) and 4.1 log_2_ lower levels of a ferrichrome-binding protein (Bc3466). In addition, a previous phenotypic study with an *rpoN* mutant, which encodes Sigma 54 in *B. cereus*, showed a pleiotropic role for this sigma factor, including the lack of motility, reduced virulence, downregulation of genes involved in the degradation of branched-chain amino acids, carbohydrate transport and metabolism [59]. Likewise, Δ*bc1009* showed significantly different expressed proteins that overlap with the affected metabolic pathways of Δ*rpoN*, including chemotaxis, virulence (Bc3104 and Bcc5239) and branched-chain amino acids (Bc1396). Interestingly, Yeak and colleagues identified the Sigma 54-dependent transcriptional activator (bc4165) to be under the regulation of *bc1009* under heat shock conditions [17]. Previously, Bartolini *et al*. [60] provided evidence for a role of the transcription factors SigB and SinR in *B. subtilis* flagella-dependent motility in liquid and semi-solid media controlling biofilm formation and dispersal. Studies of transcriptional repressor SinR in *B. thuringiensis* and *B. cereus* biofilm formation and motility were previously reported [61,62]. The *B. cereus* and *B. thuringiensis* SinR system shows several differences with that in *B. subtilis*, including the number and type of regulon members, for example, enterotoxin synthesis vs. extracellular polysaccharide production and absence of Sigma D, the sigma factor that controls chemotaxis and motility gene expression in *B. subtilis*. The underlying mechanisms, including possible interactions with Sigma B and GSR, remain to be elucidated. Lastly, the CodY transcriptional regulator was found to modulate expression of genes involved in biofilm formation, amino acid transport, metabolism, virulence and motility in *B. cereus* [63]. Deletion of the transcriptional regulator CodY resulted in reduced expression of genes related to motility and virulence, whilst genes involved in metabolism and amino acid transport were more highly expressed, which matches our Δ*bc1009* data. An initial promoter-binding motif analysis has identified a putative overlap related to RpoN/Sigma 54 between the five significantly lower expressed MCPs in *Δbc1009* and Bc1009, but future studies, including protein-DNA binding studies, are needed to confirm this.

Overall, our results show an effect on motility structure and/or regulation via the unique TCS RsbKY, methylated via RsbM, and in particular SigB-regulated Bc1009 on motility for *B. cereus* ATCC 14589, even under non-stress-inducing conditions. Mutants related to the GSR all had flagella and were motile, but trajectory analysis with Δ*bc1009* confirmed changes in the run-and-tumble motion used for *B. cereus* motility. We found a shorter duration of the run phase for Δ*bc1009*, which results in an overall lower persistence and coverage of the surface area over a given time. Impaired regulation of the run-and-tumble motion can be explained by the substantial reduction in C-ring proteins, caused by a mutation in the GSR, in addition to a reduction of a subset of MCPs modulated by Bc1009 specifically. In our newly proposed chemotaxis model, CheA and CheY are still key regulators needed for efficient run-and-tumble regulation, but an additional regulatory role for the Hpr-like protein Bc1009 in the tested conditions on modulation of MCP levels and C-ring proteins is proposed.

## Supplementary material

10.1099/mic.0.001659 Supplementary Material 1.

## Abbreviations

BHI: brain heart infusion
CCW: counterclockwise
CW: clockwise
GSR: general stress response
HBL: haemolysin BL
LCMS: Liquid Chromatography-Mass Spectrometry
LFQ: label-free quantification
MCPs: methyl-accepting chemotaxis proteins
OC: overnight culture
PPS: peptone physiological salt
SigB: sigma factor B
TCS: two-component system
TEM: transmission electron microscopy
TSB: tryptone soy broth
