# Supplementary material for: Sigma B regulated motility and chemotaxis in Bacillus cereus
Source: Microbiology (Reading). 2026 Jan 27;172(1):001659. doi: 10.1099/mic.0.001659 (PMC12848376; doi:10.1099/mic.0.001659)
Supplement: Supplementary Material 1. [file mic-172-01659-s001.pdf]

Supplementary Material files and legends

Table S1: Oligonucleotides used in this study.

Figure S2: (A) Sample trajectory color coded with respect to speed. (B) Time evolution of speed and angular velocity and detected tumbling events based on our run-and-tumble detection algorithm. (C) The detected run and tumble phases along the trajectory are indicated with gray and red colors, respectively.

Figure S3: The mean speed of *Bacillus cereus* ATCC 14579 Wild type (WT), Δ*rsbY*, Δ*bc1009* and Tn-flgG is shown during run phase (A) and tumble phase (B).

Figure S4: Surface area of *Bacillus cereus* ATCC 14579 Wild type (WT, white), five GSR-mutants (light grey), two chemotaxis mutants (dark grey) and Tn-flgG (black) on 0.25% agar TSB plates after 21 h and 48 h incubation at 30 °C. Maximum surface area is indicated by the dotted line.

Figure S5: Volcano plots of the protein abundance ratios of Δ*rsbK* (A), Δ*rsbY* (B), Δ*sigB* (C) and Δ*Bc1009* (D) over WT. Positive $\log_2$ protein abundance ratio values indicate higher abundance in the mutant compared to the WT, whilst negative values indicate a lower abundance. A cut-off of ≥ 3 $\log_2$ and the p-value of ≤ 0.05 was used and is indicated in the graphs by the black lines. Not significant proteins and significant proteins that overlapped with the Tn-flgG/WT expression pattern are indicated by a grey circle whilst significant proteins that were differently expressed from the Tn-flgG/WT expression pattern are indicated by a black circle. Different coloured circles correspond to motility-related proteins and are divided in sub-groups which are indicated in the legend. Significant motility related proteins are labelled with their KEGG protein number.

Table S6: Uniquely significant protein abundance ratio changes that are different from the expression pattern of Tn-*FlgG*/WT comparison. These proteins correspond with the black dots in the volcano plots of supplementary Figure S5. Protein ratios were considered significant if the change was ≥ 3 $\log_2$ and p ≤ 0.05. The abbreviation FB stands for fold change.

Figure S7: Bacterial chemotaxis pathway as indicated in KEGG (Jan, 2024). Proteins known to be present in *B. cereus* ATCC 1459 are indicated in green.The genes encoding CheA and CheY were deleted in the current study next to the GSR and Bc1009 encoding genes shown in Figure 4 in the manuscript, with the respective putative roles in chemotaxis explained in the text.

Table S1: Oligonucleotides used in this study	
**Oligonucleotides**	**Oligonucleotides sequence from 5' to 3'**
KO_BC1007_UP_SalI_F	GCAAGCGAGTTGTTACGCAG
KO_BC1007_UP_NotI_R	ATTATAATACTTATTTTCCACTCCAACT
KO_BC1007_down_NotI_F	ACAATAAAAAGCGTATGAAATCCTCTG
KO_BC1007_down_EcoRI_R	CTTGTATCGCTCAATTCTGCACA
BC1007_Check_F	CGGCATAGAGTGTTTAGAAATACT
BC1007_Check_R	GTTGAAAAGACAGTTATACCTTTTG
KO_BC1627_UP_SalI_F	GGTCTTATCATTATGCTCCAAAACT
KO_BC1627_UP_NotI_R	GCCATTCTTTTTAATTCTCTCCTTCTATG
KO_BC1627_down_NotI_F	GCAAATAGTTAATATAGAAGGGGT
KO_BC1627_down_EcoRI_R	GTTCAATTAGCTCCTTGTTTTGA
BC1627_Check_F	GACATACAGATAGTAGACCTATTCAT
BC1627_Check_R	CATGACGAATTTCATCTAACACA
KO_BC1628_UP_EcoRI_F	GTGATACGGAGCAAGTAGATGCA
KO_BC1628_UP_NotI_R	GCATTTTTATCCCCTACCTGTCTAAC
KO_BC1628_down_NotI_F	ACCCAGAAGGTTTATAATAGGAATGA
KO_BC1628_down_SalI_R	CAAATGGCACTGGTACTCCATCA
BC1628_Check_F	GACTTTATTGTAAAGCCATTCCAA
BC1628_Check_R	GGAACTTCGACATCTCTTGAATCA

Figure S2

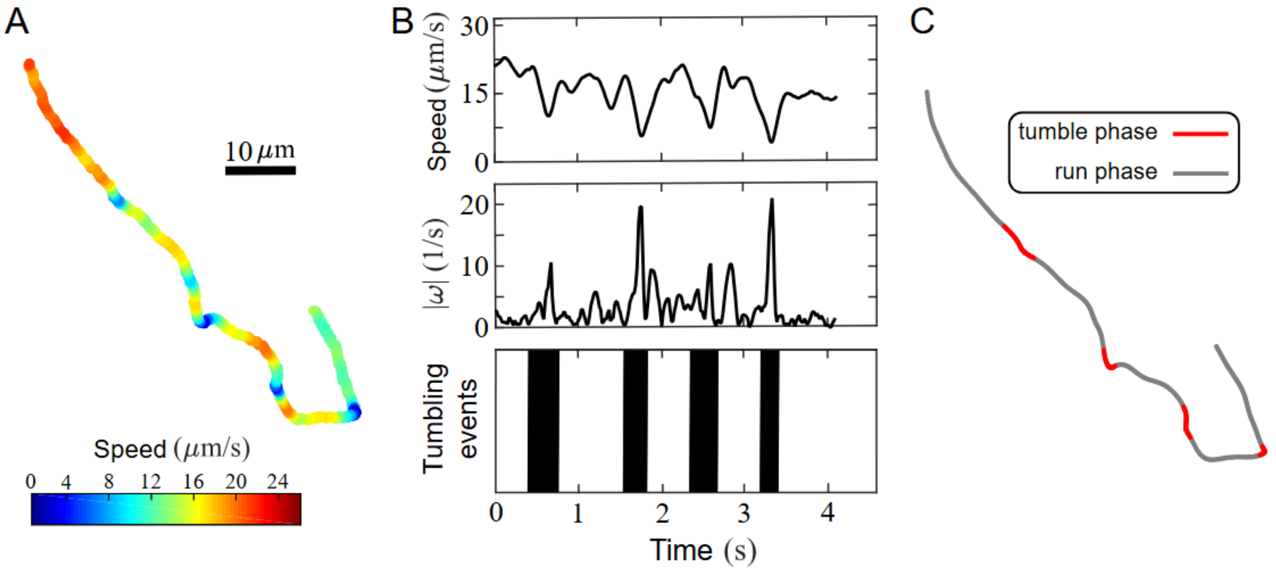

Figure S3

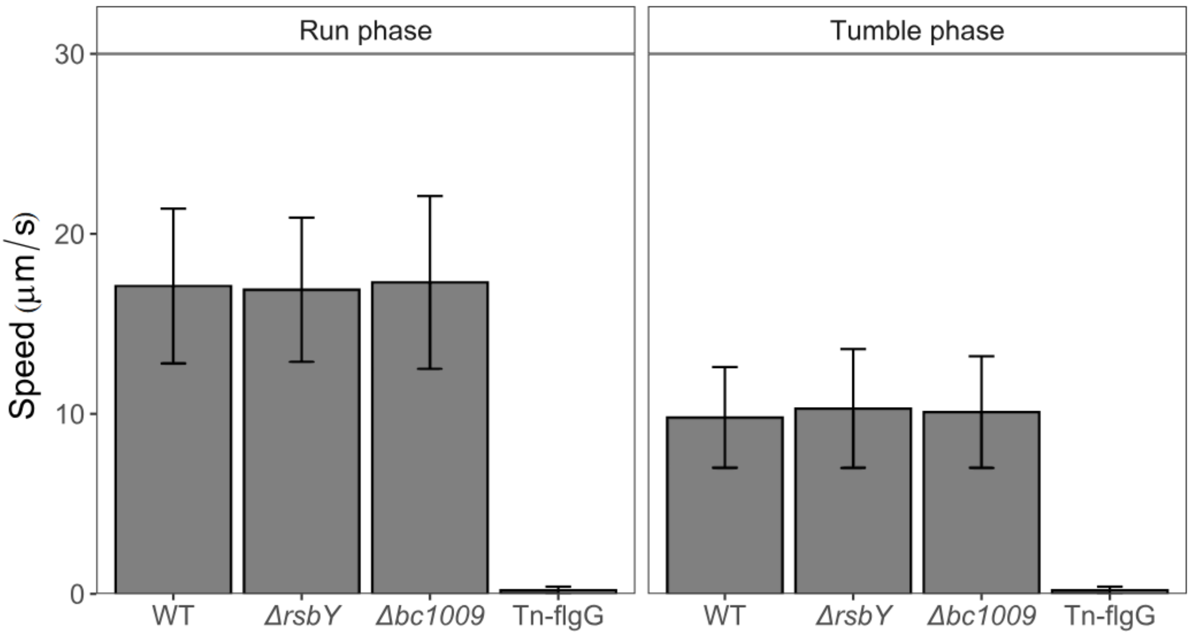

Figure S4

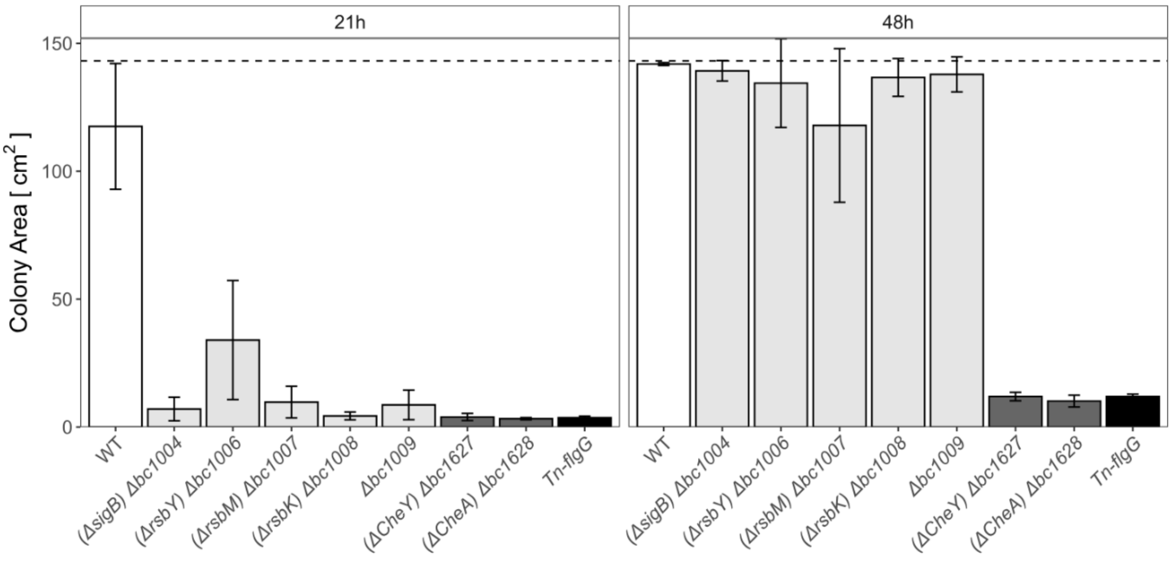

Figure S5

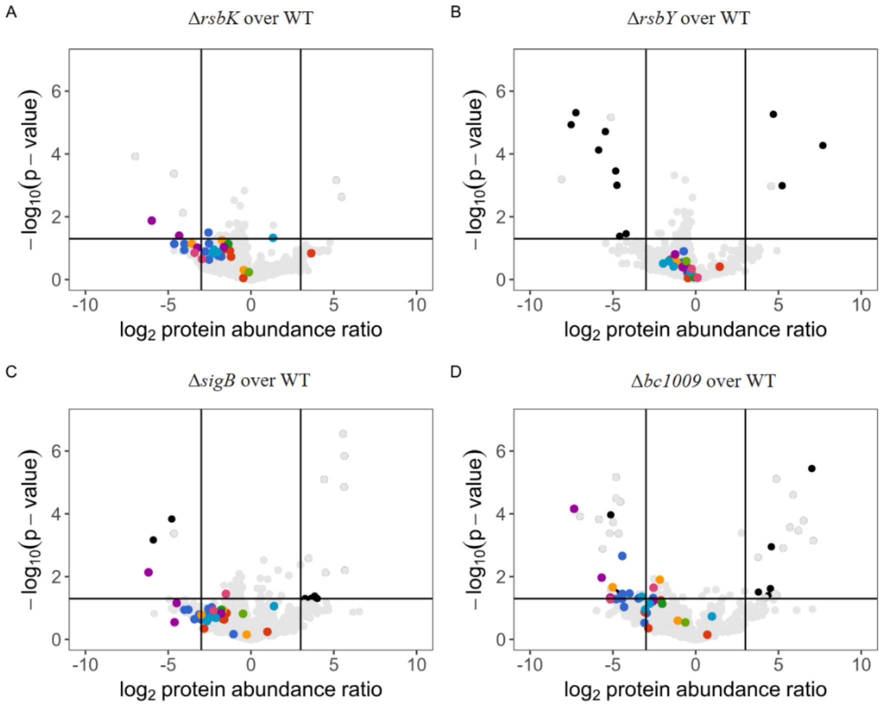

Table S6

	Protein ID	KEGG ID	Annotation	log2 FC	FC
(B) ΔrsbY/WT	Q817F5	Bc4598	FxsA protein	7.7	205.[illegible]
	Q81ES0	Bc1894	Phage protein	5.2	37.[illegible]
	Q814N6	Bc5389	Serine-type D-Ala-D-Ala carboxypeptidase	4.7	25.[illegible]
	Q81BT2	Bc3064	Permease	4.6	23.[illegible]
	Q81FA6	Bc1688	IG hypothetical 17894	-4.2	-18.[illegible]
	Q814Z3	Bc5274	UDP-N-acetylglucosamine 4,6-dehydratase	-4.6	-24.[illegible]
	Q814E2	Bcp0010	Hypothetical cytosolic protein	-4.8	-27.[illegible]
	Q814D1	Bcp0021	N-acetylmuramoyl-L-alanine amidase	-4.8	-28.[illegible]
	Q81FS9	Bc1490	Adapter protein MecA	-5.1	-34.[illegible]
	Q81EY6	Bc1822	Pyrimidine-nucleoside phosphorylase	-5.5	-43.[illegible]
	Q81CN9	Bc2713	UvrC-like protein	-5.9	-58.[illegible]
	Q81FI2	Bc1603	Cold shock protein	-7.2	-150[illegible]
	Q814F1	Bcp0001	HTH_17 domain-containing protein	-7.5	-183[illegible]
	Q814F0	Bcp0002	Uncharacterized protein	-8.1	-274[illegible]
(C) ΔsigB/WT	Q81G13	Bc1399	Ketol-acid reductoisomerase (NADP(+)) 1 (KARI 1)	4.0	16
	Q81C54	Bc2927	Prolyl endopeptidase	3.8	14.[illegible]
	Q813Q9	Bc1765	Hypothetical Cytosolic Protein	3.6	12.[illegible]
	Q81G15	Bc1396	Branched-chain-amino-acid aminotransferase	3.3	9.6
	Q812Y2	Bc3732	Hypothetical Cytosolic Protein	-4.8	-27.[illegible]
	Q81AZ9	Bc3398	Serine transporter	-5.9	-59.[illegible]
(D) ΔBc1009/WT	Q81II5	Bc0402	Cystine-binding protein	7.0	128.[illegible]
	Q81CC5	Bc2848	Oligopeptide-binding protein oppA	4.6	23.[illegible]
	Q817M3	Bc4515	Esterase	4.5	22.[illegible]
	Q81C54	Bc2927	Prolyl endopeptidase	4.4	21
	Q81G15	Bc1396	Branched-chain-amino-acid aminotransferase	3.8	13.[illegible]
	Q81AU0	Bc3466	Ferrichrome-binding protein	-4.1	-17
	Q81CN9	Bc2713	UvrC-like protein	-4.4	-20.[illegible]
	Q815C3	Bc5241	IG hypothetical 16680	-4.6	-24.[illegible]
	Q81BP7	Bc3104	Hemolysin BL lytic component L2	-4.8	-27.[illegible]
	Q815C5	Bc5239	Enterotoxin / cell-wall binding protein	-5.1	-35

Figure S7

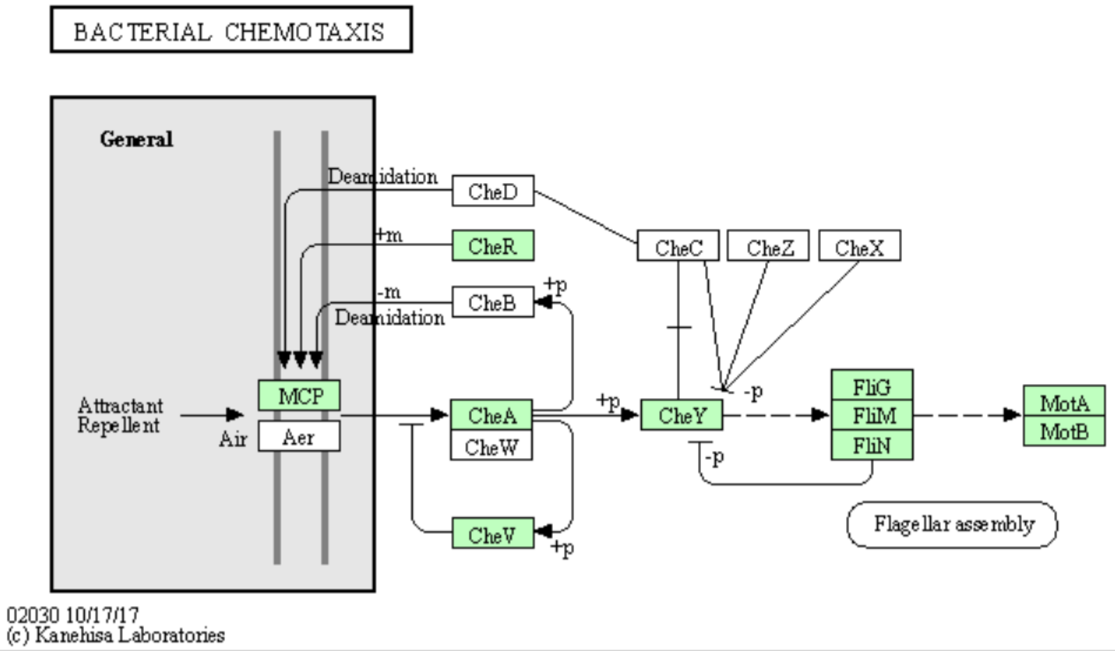

BACTERIAL CHEMOTAXIS
General
Deamidation
CheD
+m
CheR
-m
CheB
+p
Deamidation
CheC
CheZ
CheX
Attractant
Repellent
MCP
Air
Aer
CheA
CheW
+p
CheY
-p
-p
FliG
FliM
FliN
MotA
MotB
CheV
+p
Flagellar assembly
02030 10/17/17
(c) Kanehisa Laboratories
